# Association between maternal rheumatoid arthritis and small for gestational age neonates: a systematic review and meta-analysis

**DOI:** 10.3389/fpubh.2023.1075946

**Published:** 2023-09-06

**Authors:** Lv Tian, Zhiyuan Zhang, Yuting Mao, Minru Zong

**Affiliations:** ^1^Department of Rehabilitation, China-Japan Union Hospital of Jilin University, Changchun, China; ^2^School of Nursing, Jilin University, Changchun, China; ^3^Affiliated Stomatological Hospital of Zunyi Medical University, Zunyi Medical University, Zunyi, China

**Keywords:** rheumatoid arthritis, small for gestational age, systematic review, meta-analysis, maternal rheumatoid arthritis

## Abstract

**Background:**

According to reports, maternal rheumatoid arthritis (RA) has been suggested as a possible adverse factor for developing small for gestational age (SGA) in offspring. However, some studies have also indicated a need for a more statistically significant association between the two. Understanding the relationship between maternal RA and the risk of SGA is crucial for identifying potential adverse outcomes and implementing appropriate interventions. Therefore, this study aims to elucidate the association between maternal RA and the risk of offspring developing SGA.

**Methods:**

This study was registered on the International Prospective Register of Systematic Reviews (PROSPERO) (ID: CRD42022357590). A systematic literature search was conducted to identify eligible studies up to August 2022. Quality assessment was performed according to the Newcastle-Ottawa scale. The Q test and I^2^ test tested and estimated heterogeneity among studies. Odds ratios (ORs) with 95% CI were calculated using random or fixed effects models depending on the heterogeneity. Subgroup analyses, sensitivity analyses, and publication bias assessments were also performed.

**Results:**

Seven studies, including 12,323,918 participants, were included in the analysis. The results showed a statistically significant association between maternal RA and SGA (OR = 1.70, 95% CI = 1.29–2.23, *p* < 0.001). Sensitivity analysis showed stable results. The funnel plot of the symmetric distribution and the results of Begg’s and Egger’s tests showed no publication bias.

**Conclusion:**

Maternal RA is associated with an increased risk of SGA in offspring. However, more studies are still needed to explore the potential mechanisms underlying maternal RA and SGA association.

**Systematic review registration:**

https://www.crd.york.ac.uk/PROSPERO/, identifier [CRD42022357590].

## Introduction

Rheumatoid arthritis (RA)is defined as a systemic autoimmune disorder associated with a chronic inflammatory process that can damage joints and extra-articular organs, including the heart, kidneys, lungs, digestive system, eyes, skin, and nervous system (1, 2). The pathophysiological process of RA is closely related to cytokines such as interleukin 6 (IL-6) (3). Studies have shown that RA is a global disease irrespective of race, sex, ethnicity, nationality, and age, but prevalence and incidence measurements vary according to population characteristics and over time (2). Notably, gender differences have been observed in the prevalence of RA. Women are two to three times more likely to suffer from this disease than men, with most cases appearing during the childbearing years (4, 5). The global prevalence of RA is approximately 1%, fluctuates less consistently, and increases significantly from south to north, rural to metropolitan areas (6).

Small for gestational age (SGA) is defined as birth weight below the 10th percentile for gestational age (GA) (7). Perinatal mortality is higher in infants with SGA compared to newborns of appropriate gestational age at birth. This association exists for both full-term and preterm infants (8–12). SGA accounts for about 22% of neonatal deaths in low- and middle-income countries (13). It is also a cause of multiple short- and long-term complications (14). Infants with SGA are at higher risk of morbidity and developmental delay in childhood. They are susceptible to chronic diseases such as obesity, type 2 diabetes, coronary artery disease, and stroke into adulthood (15). Cytokines such as IL-6 have been reported to be higher in mothers of newborns who gave birth to SGA (16). As mentioned above, maternal RA potentially impacts SGA in offspring, but the risk of maternal RA increasing SGA in offspring is controversial. Five cohort studies of 936,030 participants found a significant association between maternal RA and risk of SGA (17–21), compared to two studies that showed no statistically significant association between maternal RA and risk of SGA (22, 23). Given the increasing incidence of RA in women over the years and the inconsistent association between maternal and risk of SGA in offspring, a Meta-analysis to assess this association would be of great value.

## Methods

### Registration information

This meta-analysis was reported according to the Preferred Reporting Items for Systematic Reviews and Meta-Analyses (PRISMA). Furthermore, it was registered on the International Prospective Register of Systematic Reviews (PROSPERO). The register number was CRD42022357590.

### Search strategy

The literature was searched from inception to August 2022 in Embase, PubMed, Web of Science, Cochrane Library, and Scopus databases. A comprehensive search strategy was conducted for articles examining maternal RA and SGA in offspring. The search strategy was based on previous systematic reviews on RA and SGA. The complete retrieval strategy was finalized in discussions among three authors (Lv Tian, Zhiyuan Zhang, and Yuting Mao). The search strategy for this study consisted of three components: rheumatoid arthritis, pregnant mothers, and small for gestational age. Each component was searched using a combination of free and subject terms, and the components were combined using specific database terms. The search strategy for PubMed is shown below. It was adapted for the other databases (((((((Arthritis) OR (Rheumatoid)) OR (Rheumatoid Arthritis)) OR (Rheumatoid Nodule)) OR (Rheumatoid Vasculitis)) OR (“Arthritis, Rheumatoid”[Mesh])) AND ((“Infant, Small for Gestational Age”[Mesh]) OR (“small for gestational age”))) AND (Maternal).

### Selection criteria

The inclusion criteria developed for screening eligible publications were as follows:

1. The type of study was a case–control study or cohort study.
2. The outcome of interest was the risk in SGA.
3. Estimates such as ratio ratios (ORs) or relative risks (RRs) with the corresponding 95% confidence intervals (CIs) were reported, or sufficient data were provided to perform the calculation.
4. Pregnant women with no clear indication of special treatment (biologic therapy).

Exclusion criteria were as follows:

1. The study was not in humans, e.g., in vitro and in vivo studies.
2. The article was in the review category.
3. Risk estimates could not be calculated.
4. Duplication.

### Data extraction and quality assessment

Two participants (Lv Tian and Zhiyuan Zhang)extracted the data independently according to a predefined data form and then assessed the quality of the articles. If disagreements were encountered, discussions were held to reach an agreement, or a third person (Yuting Mao) was consulted regarding recommendations. Data extracted included authors’ names, year, country, study design, comparisons, total participators, OR or RR with 95% CI, quality, adjustment factors, assessment method, and age of the study participators. The quality of observational studies was assessed by the Newcastle-Ottawa scale, which has three columns: selection, comparability, and outcome/exposure. The total score is nine stars, with six or more stars for high-quality literature and 4–5 stars for moderate quality (24).

### Statistical analysis

The association between maternal RA and SGA in offspring was assessed by pooling the ORs with the corresponding 95% CIs. RRs were transformed into odds ratios which yielded similar estimates as ORs. the formula, RR = OR/[(1 − P0) + (P0 × OR)], was used to perform RR to OR conversion (P0 represents the incidence of the outcome in the non-exposed group) (25). When multiple ORs were provided in a single study, the one that controlled the most confounders was selected. Heterogeneity between studies was tested by Q-test at the *p* = 0.1 level and then based on I^2^ statistics: I^2^ < 50% of respondents indicated no significant statistical heterogeneity and therefore a fixed effects model was selected, I^2^ > 50% indicated statistical heterogeneity, and a random effects model was used (26, 27). Sensitivity analyses were achieved by the leave-one-out method (28). Publication bias was assessed by observing whether the funnel plot was symmetrical and calculating Begg’s and Egger’s test values. Data were processed using the statistical software Stata 15.0 (Stata Corp., College Station, TX, United States) (29, 30). *p* values less than 0.05 were considered statistically significant.

## Results

### Research characteristics

**Figure 1:**
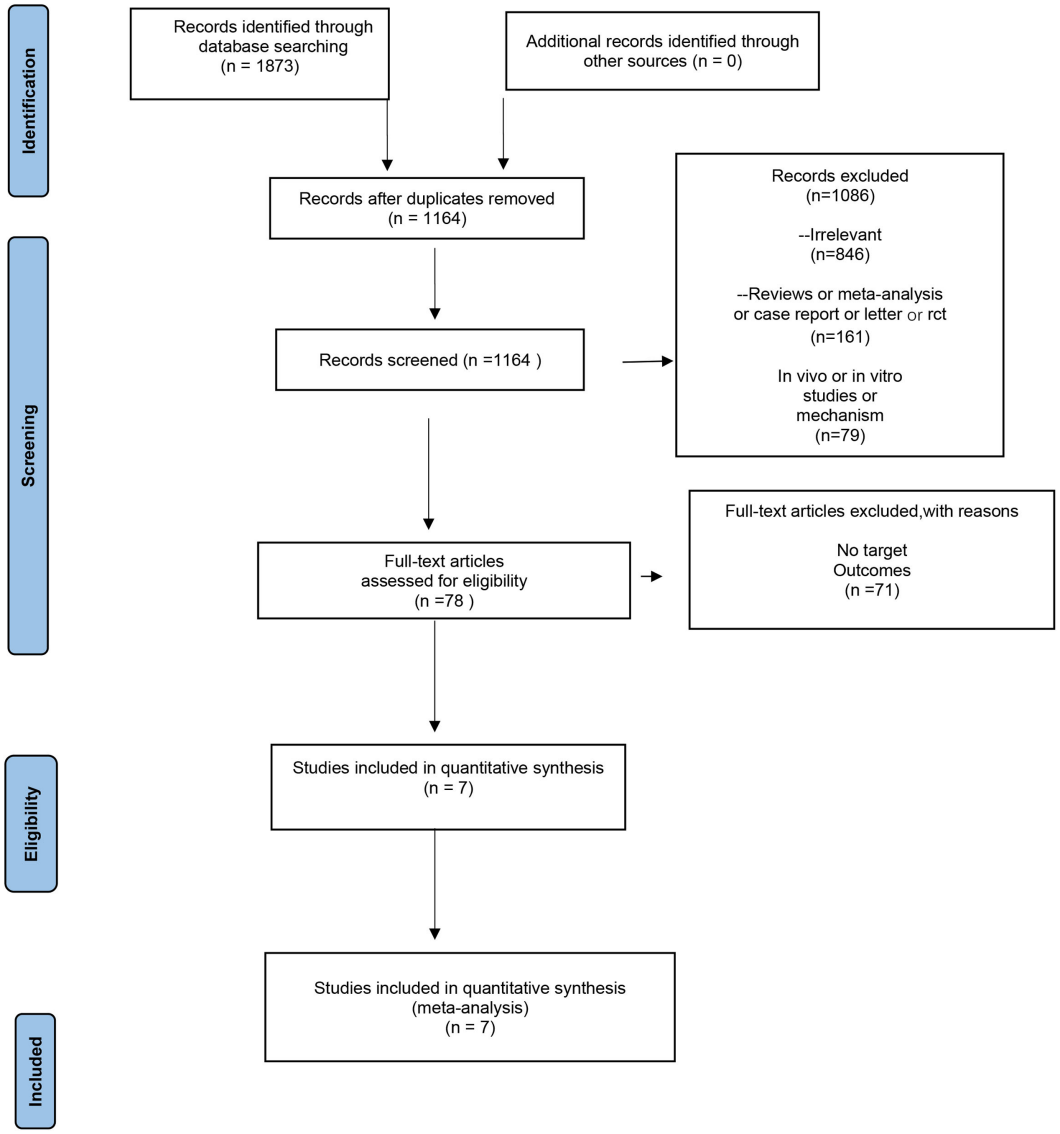
Preferred reporting items for systematic reviews and meta-analysis (PRISMA) flow chart.

According to the search strategy, the initial electronic search yielded 1872 articles, and 1,164 articles were obtained after eliminating duplicates. Seventy-eight articles were retained after reading the titles and abstracts. Finally, the full text was read through to filter the literature, and finally, seven articles were obtained for inclusion (17–23). The detailed literature screening process is shown in Figure 1.

This meta-analysis included seven articles involving a total of 12,323,918 participants, published between 2006 and 2019. Of these, three were conducted in the United States, two in Canada; one was conducted in Norway, and one in Switzerland. Six of them were adjusted for confounding factors. All included studies were of high or moderate quality. Table 1 presents more detailed information on the included studies.

**Table 1:** Characteristics of individual studies included in the meta-analysis.

Author (year)	Study design (retrospective)	Comparison	Country	Sample size	Age of the study participators	Assessment method	OR (95%CI)	RR (95%CI)	Quality	Adjustment factors
Hissah Aljary (17)	Cohort	Fetal/neonatal outcomes in patients with RA vs. patients without RA	United States	8,417,607	No limit	RA:ICD-9-CM	2.36 (2.09–2.66)		Moderate	Adjusted^a^
Cheryl Barnabe (18)	Cohort	Neonates of women with RA vs. neonates of RA controls	Canada	188	The mean age of women with RA was 32 years (standard deviation (SD) 5.5, range 21–42)	RA:ICD-9-CM	2.99 (1.27–7.04)		High	Unadjusted
Stephanie O. Keeling (19)	Cohort	Women with RA vs. women without IA	Alberta, Canada	312,081	≤54 years old	RA:ICD-10 CA	1.51 (1.21–1.88)		High	Adjusted^b^
Susan D. Reed (22)	Cohort	Women with RA vs. women without RA	Washington State, United States	2,842	No limit	RA:ICD-9	1.56 (0.94–2.68)	1.51 (0.94–2.43)	High	Adjusted^c^
Jennifer Strouse (20)	Cohort	Women with an ARD vs. women without a recorded ARD	California, United States	2,963,888	No limit	RA:ICD-9-CM	1.49 (1.36–1.63)		High	Adjusted^d^
Marianne Wallenius (23)	Cohort	Women with RA vs. women with reference deliveries	Norway	627,138	No limit	RA:ICD-10CA	1.10 (0.90–1.40)		High	Adjusted^c^
Astrid Zbinden (21)	Cohort	Women with RA vs. women with axSpA	Switzerland	244	No limit	Patients with RA had to fulfil the revised 1987 ACR classification criteria for RA. Patients with axSpA fulfilled the ESSG criteria for SpA.	13 (1.67–100.63)		High	Adjusted^e^

a Adjusted for age, race, income and type of insurance.

b Adjusted for the following: age at delivery, rural residence, ethnicity, 2010 annual household income, nulliparity, renal disease, hypertension, hypertensive disorders in pregnancy, diabetes, gestational diabetes, cerebrovascular disease, ischemic heart disease, and hyper/hypothyroidism.

c Adjusted for maternal age, smoking, and delivery year.

d The expression is vague.

e Adjusted odds ratio for maternal age and gravidity.

ICD-9-CM, international classification of diseases, ninth revision, clinical modification; ICD-10-CA, international classification of diseases, tenth revision, canadian enhancement; RA, rheumatoid arthritis; IA, inflammatory arthritis; ARD, autoimmune rheumatic disease; axSpA, axialspondyloarthritis.

### Overall meta-analysis

**Figure 2:**
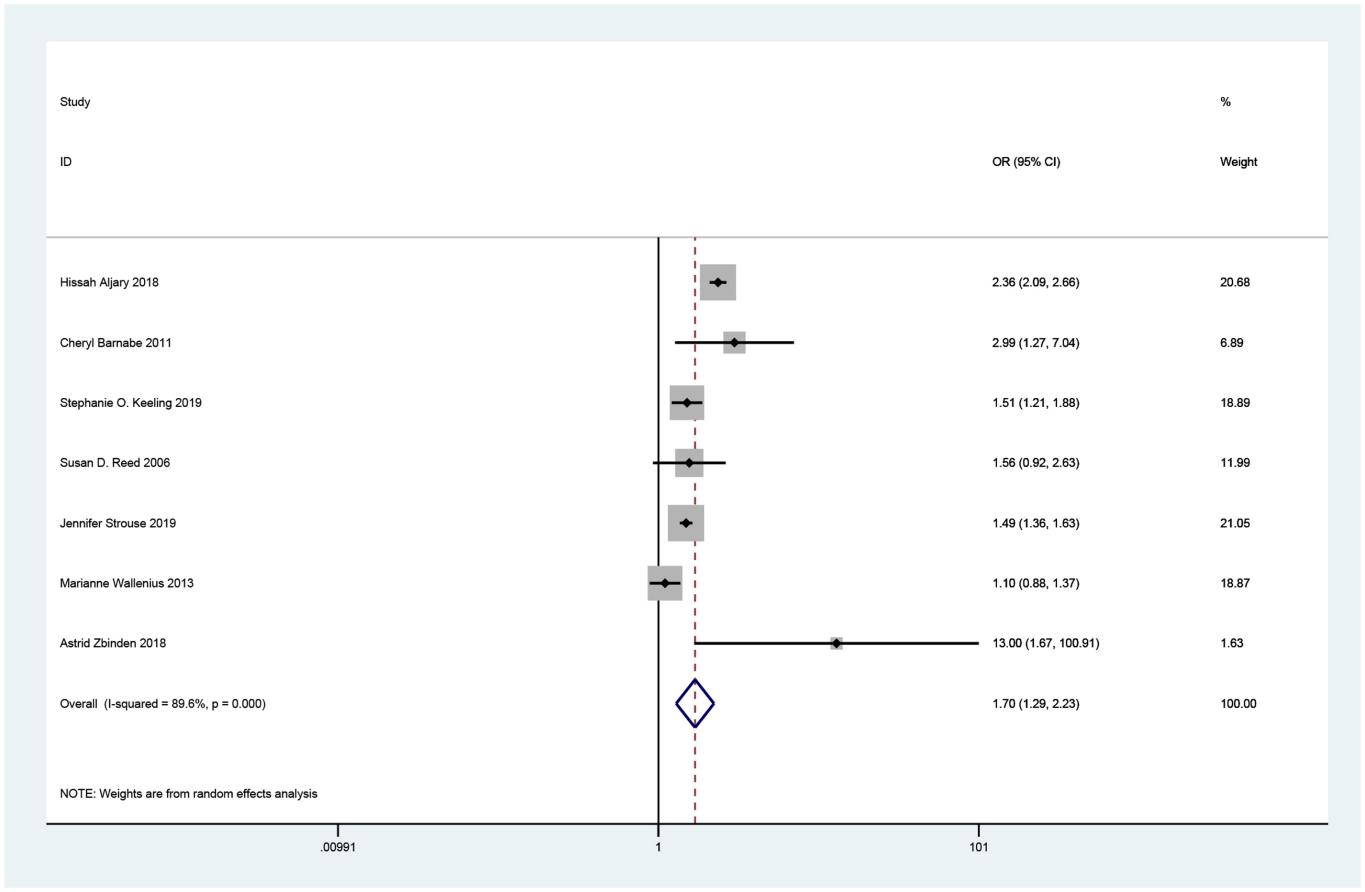
Forest plot of the association between maternal rheumatoid arthritis and small for gestational age neonates.

Seven studies related to the association between maternal RA and the risk of SGA in offspring were included in the overall meta-analysis (17–23). Heterogeneity was observed, and a random effects model was used to combine the studies. Pooled results showed that maternal RA was statistically significantly associated with increased risk of SGA in offspring (OR = 1.70, 95% CI = 1.29–2.23, *p < 0.001*; I^2^ = 89.6%; Figure 2).

### Subgroup analyses

In subgroup analyses, according to the country of study, three studies were conducted in the USA (17, 20, 22) (OR = 1.80, 95% CI =1.23–2.63, *p = 0.003*). Two studies conducted in Canada (18, 19) (OR = 1.87, 95% CI =1.01,3.46, *p = 0.048*) showed a statistically significant association between maternal RA and increased risk of SGA in offspring. A study from Norway reported an OR of 1.1 (95% CI = 0.88–1.37), and a study from Switzerland reported an OR of 13.0 (95% CI =1.68–100.91). For adjustment factors, a significant association was observed in adjusted studies (OR = 1.63, 95% CI =1.23–2.16, *p = 0.001*). For subgroup analyses with different study qualities, the results remained unchanged [high quality (18–23): OR = 1.47 95% CI =1.20–1.80, *p < 0.001*]. For the age of women with RA, the results remained unchanged in the two age-restricted studies and the five studies with unrestricted age [unrestricted (17, 20, 22, 23) OR = 1.67, 95% CI =1.78–2.35, *p = 0.004*, restricted (18, 19) OR = 1.87,95% CI = 1.01–3.46, *p = 0.048*]. All these results are shown in Table 2.

**Table 2:** Summary of pooled ORs with CI in the meta-analysis.

			Heterogeneity		Significant		
Analysis	No. of studies	OR (95% CI)	*p*	I^2^ (%)	*Z*	*p*	*M* ^a^
Overall	7	1.70 (1.29,2.23)	<0.001	89.6%	3.80	<0.001	R^c^
Country							
United States	3	1.80 (1.23,2.63)	<0.001	94.4%	3.02	0.003	R^c^
Canada	2	1.87 (1.01,3.46)	0.130	56.4%	1.97	0.048	R^c^
Norway	1	1.1 (0.88,1.37)	NA	NA	NA	NA	NA
Switzerland	1	13.0 (1.68,100.91)	NA	NA	NA	NA	NA
Adjustment factors							
Adjusted	6	1.63 (1.23,2.16)	<0.001	91.1%	3.38	0.001	R^c^
Unadjusted	1	2.99 (1.27,7.04)	NA	NA	NA	NA	NA
Quality							
High	6	1.47 (1.20,1.80)	0.018	63.5%	3.71	<0.001	R^c^
Moderate	1	2.36 (2.09,2.66)	NA	NA	NA	NA	NA
Age of the study subjects							
No limit	5	1.67 (1.18,2.35)	<0.001	92.8%	2.91	0.004	R^c^
Limit	2	1.87 (1.01,3.46)	0.130	56.4%	1.97	0.048	R^c^
Sample size							
A	2	1.87 (1.19,2.94)	<0.001	97.2%	2.73	0.006	R^c^
B	5	1.57 (1.11,2.21)	0.013	68.2%	2.56	0.011	R^c^

^a^Model of meta-analysis, ^b^fixed effects model, ^C^random effects model; NA, not applicable. A: more than one million, B: less than one million, OR, odds ratio.

### Sensitivity analyses and publication bias

**Figure 3:**
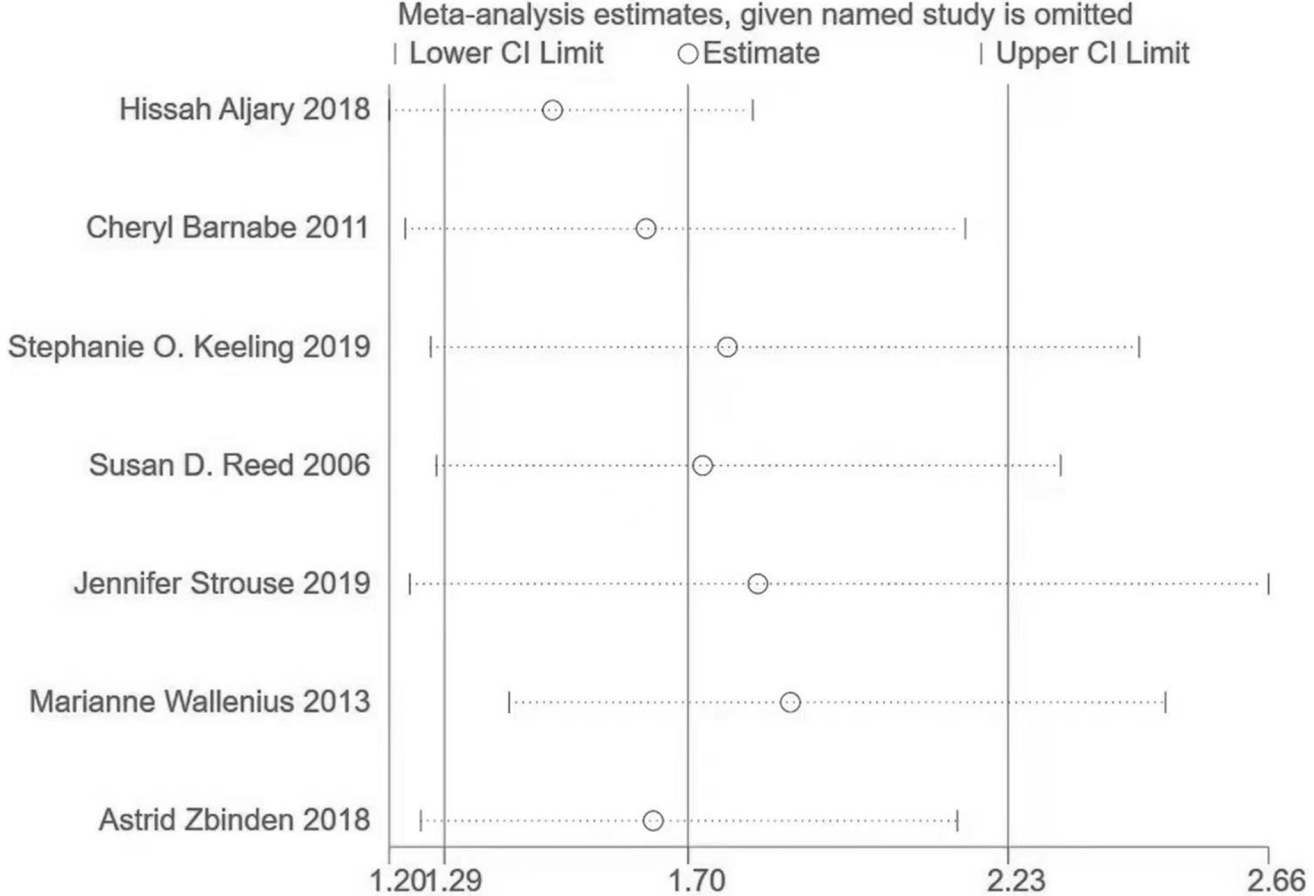
Sensitivity analyses.

**Figure 4:**
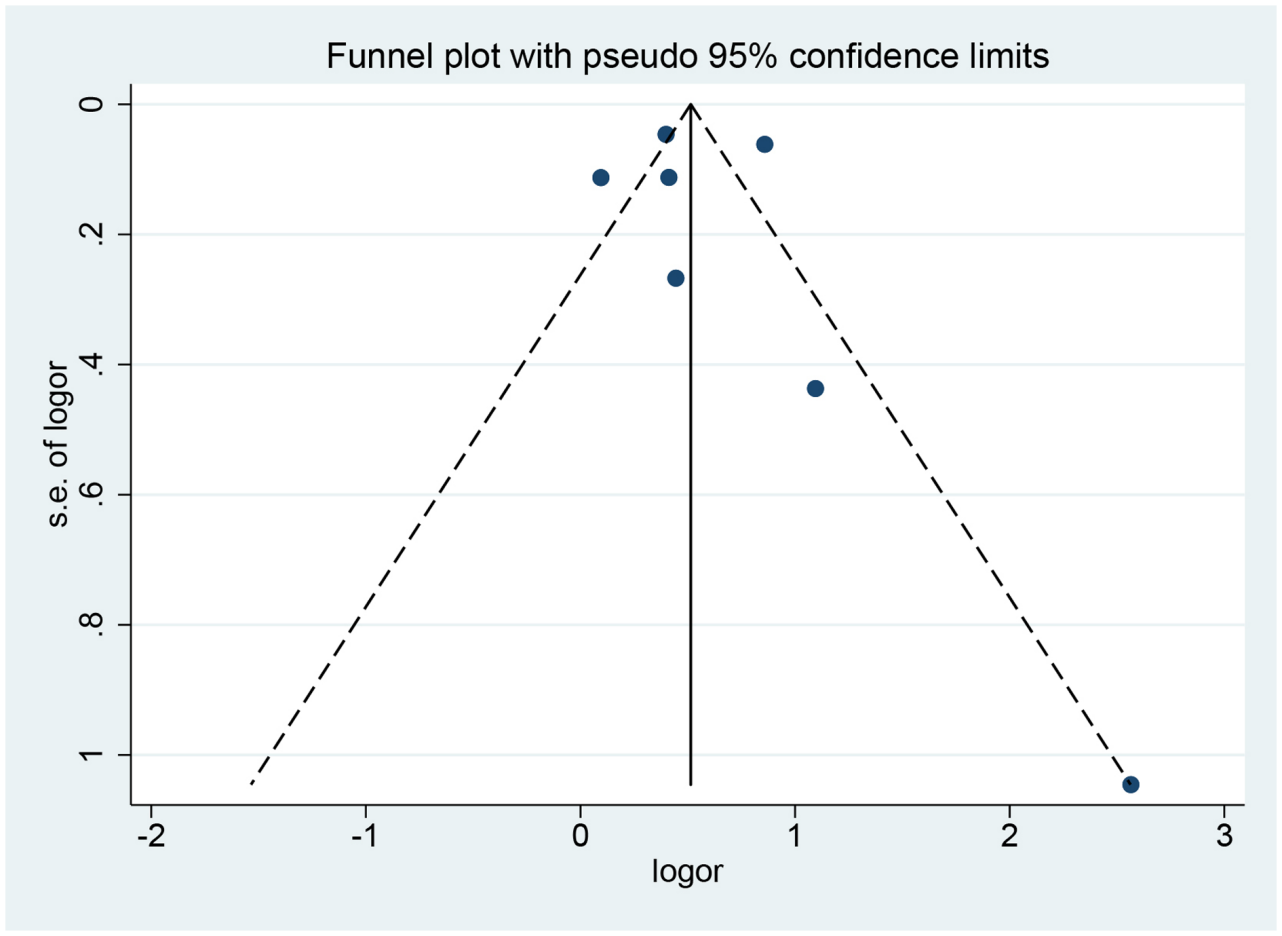
Funnel plot (potential publication bias of included studies).

Sensitivity analyses were performed using the leave-one-out method, which indicated that the result was stable (Figure 3). The funnel plot was symmetrically distributed, and Begg’s test and Egger’s test showed no publication bias (Begg’s test: *Z* = 0.90; *p* = 0.386; Egger’s test: *t* = 0.27; *p* = 0.795; Figure 4).

## Discussion

The association between maternal RA and the risk of SGA in offspring is a controversial topic. Based on previous studies, this meta-analysis of seven cohort studies of patients from different backgrounds found an association between maternal RA and increased risk of SGA in offspring. The results show that newborns of women with RA are more likely to be SGA compared to women with normal pregnancies. In addition, subgroup analyses confirmed the such association. The pooled estimated effects of the included subgroups were almost similar to the overall results. Statistical significance was found for subgroups grouped by country, study quality, age with or without restrictions, sample size, and adjustment factors. Sensitivity analysis showed no substantial change in the results when we used the retention method, suggesting that the results of this meta-analysis are robust. Considering the progressive increase in the prevalence of RA among women, this article will make much sense.

The potential mechanisms between maternal RA and SGA of the newborn remain obscure. It has been shown that oxidative stress markers are significantly higher in neonates with SGA than in infants of appropriate gestational age (AGA) and that prolonged excessive oxidative stress and intrauterine malnutrition can lead to SGA in offspring (31). Oxidative stress plays a crucial role in the pathophysiology of RA. In RA patients, the antioxidant system is altered, and serum and synovial fluid lipid peroxidation levels are elevated (32). Since the last decade, studies have shown a positive correlation between the level of ROS and the severity of RA (33). The findings suggest that maternal RA can affect SGA in offspring through reactive oxygen species.

In another study, IL-10 was found to be detectable in 23.4% of RA patients and IL-6 in 76.6% of patients in late pregnancy. When IL-6 levels were high, the risk of neonatal SGA was increased, suggesting that increased levels of IL-6 are associated with neonatal SGA (16). In addition, vitamin D is also involved. Vitamin D deficiency increases levels of pro-inflammatory cytokines and leads to oxidative stress. Lower 25(OH)D status is associated with increased vascular endothelial cell expression of interleukin six and reduced expression of vitamin D receptors and 1-alpha hydroxylase (34). One study reported significantly higher levels of pro-inflammatory cytokines in the cord blood of SGA infants than in the cord blood of non-SGA infants (35). Maternal rheumatoid can increase the risk of SGA in newborns through cytokines. In clinical studies, patients with RA were found to have low sleep quality and severe sleep disturbances (36). A short duration of pregnancy and poor sleep quality were associated with an increased risk of preterm birth and SGA (37). This result supports the effect of maternal rheumatoid on neonatal SGA. Our study confirms the results, but more profound and more comprehensive mechanisms remain to be explored further.

This meta-analysis has several limitations. First, heterogeneity existed between included studies, so a random effects model was applied to combine ORs and RRs. Although subgroup analysis could explain some of the heterogeneity, sufficient data from the included original studies, such as different ethnicities of female RA patients and different rheumatoid disease characteristics (duration, severity, with or without treatment), were lacking to investigate other potential sources. Second, although the sensitivity analysis results were robust and ensured the accuracy and persuasiveness of the results, the limited number of studies conducted outside of Europe and the Americas suggests the need for more data on populations of different backgrounds. The substantial heterogeneity (I^2^ = 89.6%) of the pooled effects may reflect the significant variation in sample size. Considering the limited number of studies in our review and the high heterogeneity, more and higher quality studies are needed. Our results should be interpreted cautiously due to the limited number of included studies. Finally, our study is the absence of correlation analysis between maternal RA disease activity and the risk of neonatal SGA. Unfortunately, we could not investigate this potential association due to the need for more sufficient data in the included studies. However, we acknowledge this limitation and encourage future research to explore the relationship between maternal RA disease activity and the risk of SGA. Despite these limitations, the following strengths of this study should be acknowledged. This is the first meta-analysis to elucidate the relationship between maternal RA and the risk of SGA in offspring. In addition, sensitivity analyses showed that the meta-analysis results did not change substantially using the leave-one-out method. The results were robust, and there was no publication bias.

Accurate knowledge of maternal RA and neonatal SGA risk is valuable for early intervention to reduce the frequency of these adverse events. Further studies are needed to investigate the underlying causes of RA and SGA, which may improve clinical care and lead to a more informed prognosis for future pregnant mothers with RA.

## Conclusion

In summary, maternal RA increases the risk of SGA in offspring. Healthcare providers should be aware of the high risk of adverse maternal and neonatal outcomes. Medical staff should develop an individualized and continuous pregnancy screening program for pregnant women with RA, record their family history, medical history, and physical examination data in detail, and provide health education on daily precautions for pregnant women. Before pregnancy, women with RA should be encouraged to maintain optimal health and manage their RA and other chronic diseases. Pregnant women with RA should schedule detailed and regular pregnancy tests according to the actual situation. By increasing the number of pregnancy tests, we can dynamically monitor the mother and fetus’s physiological changes and effectively reduce the risk of SGA.

## Data availability statement

The original contributions presented in the study are included in the article/supplementary material, further inquiries can be directed to the corresponding author.

## Author contributions

LT: data curation, formal analysis, and writing original draft. ZZ: investigation. YM: resources and supervision. MZ: project administration, visualization, writing-review, and editing. All authors contributed to the article and approved the submitted version.

## Funding

This study was supported by the Natural Science Foundation of Jilin Province (grant number: YDZJ202201ZYTS062, 20200201127JC).

## Conflict of interest

The authors declare that the research was conducted in the absence of any commercial or financial relationships that could be construed as a potential conflict of interest.

## Publisher’s note

All claims expressed in this article are solely those of the authors and do not necessarily represent those of their affiliated organizations, or those of the publisher, the editors and the reviewers. Any product that may be evaluated in this article, or claim that may be made by its manufacturer, is not guaranteed or endorsed by the publisher.
